# Risk Factors of Underweight in Children Aged 6–59 Months in Ethiopia

**DOI:** 10.1155/2017/6368746

**Published:** 2017-11-13

**Authors:** Deneke Tosheno, Yohannes Mehretie Adinew, Thilagavathi Thangavel, Shimelash Bitew Workie

**Affiliations:** ^1^Wolaita Zonal Health Department, Sodo, Ethiopia; ^2^College of Health Sciences and Medicine, Wolaita Sodo University, Sodo, Ethiopia

## Abstract

**Background:**

Undernutrition in early childhood has irreversible and long-lasting implications. Hence, this study was aimed at assessing risk factors of child undernutrition.

**Methods:**

A community-based cross-sectional study was conducted on 642 households with mothers to children pairs aged 6–59 months selected by a multistage systematic random sampling method. Child anthropometric measurements on weight were recorded using standardized and calibrated weighing scales. Weight-for-age was compared to the 2007 WHO growth reference by WHO Anthro software. Data were entered using Epi-Info and analyzed using SPSS. Bivariate and multivariate logistic regression analyses were used to evaluate the association between underweight children and their predictors; both crude and adjusted odds ratios with 95% confidence interval were reported.

**Results:**

One-fourth (25%) of the children were underweight. Child age (AOR: 2.36), gender (AOR: 1.82), illness (AOR: 0.09), maternal decision making power (AOR: 0.07), maternal education (AOR: 0.19), employment/occupation (AOR: 5.29), and household income (AOR: 4.16) were found to be independent and significant predictors of underweight children.

**Conclusion:**

Significant proportion of the children were underweight. Maternal decision-making power persists as a strong predictor of children's weight. Therefore, intervention programs focusing on improving mothers' decision-making power on child nutrition would contribute to the efforts towards alleviating the problem.

## 1. Background

Undernutrition is still a prevailing evil affecting under-five children in the developing world [1]. Its consequences are severe and have irreversible and long-lasting implications [2]. It is responsible for the annual deaths of 3.5 million under-five children globally and the third disease burden of this age group. It continues to be a significant global public health and development concern [3].

Globally, an estimated 16% of under-five children were underweight (i.e., weight-for-age below –2 SD) in 2011. The estimated prevalence of underweight in 2011 was 17.7% in Africa, 21.4% in Sub-Saharan Africa, and 19.3% in East Africa [4, 5]. More than 25% of under-five children in the developing world are malnourished. Among these malnourished children, nearly three-quarters live in just 10 countries of Sub-Saharan Africa region; Ethiopia and Nigeria alone share 33% of the region's burden [6].

In Ethiopia, large proportions of children have been suffering from undernutrition. According to the 2014 Ethiopian Mini Demographic and Health Survey (EDHS) [7], 25% of under-five children were underweight and among those 7% were severely underweight. As to the south region, the prevalence of underweight is comparable to the national level with 25.7% underweight and 7.9% severe underweight. This shows that child malnutrition is still a critical problem in Ethiopia.

Moreover, in identifying causes and determinants of child underweight, little research has addressed the association between women's decision-making power and child underweight, which reflects both cumulative and acute exposures to malnutrition in Ethiopia in general and as a study-setting in particular. Therefore, this study focuses on examining the association between children's underweight and mothers' decision-making power.

## 2. Methods and Materials

### 2.1. Study Design and Area

A community-based cross-sectional study was conducted in Wolaita Sodo town. Wolaita Sodo is located 385 km to the south of the capital Addis Ababa. According to the 2007 Ethiopian national population and housing census, the population of the town was projected to be about 137,522 in July 2014, with a 1.03 : 1 male to female ratio [8]. The town was structured into 3 sub-cities and 11 kebeles (the smallest administrative unit). There were 2 hospitals, 3 health centers, 11 health posts, and 21 private clinics. The main staple food of the population was teff and maize.

### 2.2. Source Population and Sample Size Determination

Source population was all children aged 6–59 months and their mothers in Wolaita Sodo town. All selected children aged 6–59 months and their mothers living in selected kebeles of Wolaita Sodo town were study population. Sample size was determined by using a single population proportion formula with the assumptions of 95% CI, 5% of margin of error, reported prevalence of underweight among under-five children in the southern region to be 25.7% [5], design effect 2, and 10% possible nonresponse rate, which gives the total sample size of 642.

### 2.3. Inclusion and Exclusion Criteria

All children aged 6–59 months and their mothers who were permanent residents of selected kebeles and households were included. Children who were seriously ill or had edema and physical deformities of limbs and spines were excluded because of difficulty in anthropometric measurements. Children whose mothers were severely sick and unable to respond were also excluded.

### 2.4. Sampling Technique

A multistage systematic random sampling technique was used. First, out of 11 kebeles in Sodo town, 5 were randomly selected. Data from the town health offices and health posts on a number of households with children aged 6–59 months were identified for the randomly selected kebeles. The total sample size was proportionally allocated to the size of children aged 6–59 months in the selected kebeles. Systematic random sampling technique was employed to select the households/mothers and children aged 6–59 months enrolled in the study. Sampling interval was calculated by dividing total study population in the selected kebeles by the number of sample units (*N/n*), which was 6760/642 ≈ 10. The first household was selected within the sampling interval of (1–10) by drawing a random number. Then the sample was taken from every 10th house of the selected kebeles until the required sample size was obtained.

### 2.5. Variables Measurement and Definition

#### 2.5.1. Dependent Variable

Children with *Z* score of less than −2 were categorized underweight, whereas those with −2 or above were categorized normal as per WHO *z* score.

#### 2.5.2. Independent Variables

Socioeconomic and demographic factors sex, age, family size, monthly average income, maternal education, decision-making power, occupation and marital status, child dietary intake, and health related disease factors were among the independent variables. Mothers' decision-making power in the household was measured by asking them eight items. For each item, the response was scored 2 if a woman made a sole decision, 1 if she was involved with a husband/partner or someone else, and 0 for otherwise. The sum of the scores was made to represent an overall index of a woman's decision-making power as indicated by previous studies [9, 10]. The total maximum score of decision-making power was 16 and the minimum was 0. Hence, those women who scored eight and above were categorized as women with high decision-making power, whereas those who scored less than eight were categorized as women with low decision-making power.

### 2.6. Data Collection and Quality Control

Pre-tested structured questionnaire was used for interviews, and a digital portable calibrated SECA weighing scale was used for anthropometric measurements. Data were collected by eight experienced diploma-holding nurses. Two senior nurses supervised the data collection.

Weight of children was measured by using the standard digital SECA scale to the nearest 0.1 kg. For weight measurement study, participants removed their shoes and jackets and wore light clothing. For children less than 2 years old and who did not stand still or those who jumped, tarred weighing was done. The age of the children was confirmed from the birth certificate or the child's immunization card, if any, or by asking the mother. If the mother could not remember the child's age, it was probed and local events were used to assist the mother.

A two-day-training was given to data collectors and supervisors on the tool, interviewing skills, and the use of weight scale, in order to minimize inter- and intraobserver errors. Technical error of measurement (TEM) was computed during training. For this, an expert took two weight measurements of ten children and let the data collectors take the measurements of all ten children twice. Then, the data was entered and computed by ENA SMART software and was confirmed acceptable. Weighing scale was calibrated to zero before taking every measurement. Measurement of weight was taken in duplicate for each child. The questionnaire was pretested in 5% of households selected from kebeles not included in the study, and some modifications were made based on response categories. To improve the quality of data, data collectors were closely supervised; each completed questionnaire was checked to ascertain all questions were properly filled and corrected by a principal investigator on daily basis.

### 2.7. Data Processing and Analysis

Data were coded and entered into Epi-Info version 3.5.4 and WHO Anthro version 3.2.2 software and exported to SPSS version 20 for further analysis. Continuous variables were checked for normality using Kolmogorov-Smirnov test. Descriptive statistics were done. Hosmer-Lemeshow test was performed for model fitness, and multicollinearity was also checked using variance inflation factor and correlation coefficients. Bivariate and multivariate logistic regression analyses were used to evaluate the association between children's underweight and predictor variables; adjusted odds ratios with 95% confidence interval were reported. Statistical significance was considered at *P* value less than 0.05.

## 3. Results

### 3.1. Sociodemographic Characteristics

A total of 642 (100.0%) questionnaires were eligible for analysis. Nearly two-thirds, 437 (68.4%), of the children were in the age category of 25–34 months and 51.9% of them were boys. The majority of the respondents, 386 (60.1%) and 463 (72.1%), were protestant in religion and Wolaita in their ethnicity, respectively. About 85% of the respondents had formal education, 306 (47.7%) were housewives, and 615 (95.8%) were married. Nearly half, 304, of them (47.4%) had less than five as a family size. Forty two percent of the respondents had less than 2000-birr average monthly income (Table 1).

### 3.2. Health, Feeding Practice, and Dietary Intake of Children and Prevalence of Underweight

Mean dietary diversity score for study participants was 5.05. About 132 (20.6%) of study participants had a poor dietary diversity (DDS ≤ 3) and another 380 (59.2%) had a medium dietary diversity score (DDS 4–6), whereas 130 (20.2%) of study participants had a good dietary diversity score, DDS ≥ 7. The majority, 513 (79.9%), of the mothers had timely initiated complementary food for their children. Only 117 (18.2%) children experienced sickness during the last two weeks prior to data collection. Prevalence of the underweight was found to be 158 (24.6%) with 95% CI (21.3%, 27.9%), 126 (19.6%) moderate, and 32 (5.0%) severe. The prevalence of underweight was more in male children 92 (27.6%) compared to their female counterparts, 66 (21.4%) (Table 2).

### 3.3. Household Decision-Making Power/Autonomy of Mothers

Among the eight decision-making items, the sole involvement of women was more in foods to be given to children, 401 (62.5%), and the purchase of small household needs, 272 (42.4%). Women hold very low decision-making power in large household purchases, 44 (6.9%). More than half of the women had joint involvement regarding large household purchases, 325 (50.6%), child health care, 361 (56.2%), using contraceptive methods, 505 (78.7%), participating in money earning works, 440 (68.5%), and when and where to take sick child leave for treatment, 420 (65.4%). Decision-making index showed that 71.1% of women had high decision-making power on the household decisions (Table 3).

### 3.4. Factors Associated with Underweight

In multivariate logistic analysis, children of 24–45 months age group were 2.36 times (AOR = 2.36, 95% CI (1.03–5.4)) more likely to be underweight compared to children who were in an age group of 48–59 months. Male children were 1.8 times (AOR = 1.82, 95% CI (1.07–3.09)) more likely to be underweight than females. Children whose mothers had only secondary education were 81% more (AOR = 0.19, 95% CI (0.05–0.73)) likely to be underweight than those whose mothers had diplomas and above. Children whose mothers' occupations were housewives were 3.26 times (AOR = 3.26, 95% CI (1.06–9.99)) more likely to be underweight than those of employees. Children from families whose monthly income was 4000.00 ETB and below were about four times (AOR = 4.16, 95% CI (1.57–11.05)) more likely to be underweight than those who had above 4000.00 ETB. Children who did not experience illness for the last two weeks prior to the survey were 91% less likely to be underweight than those who were sick (AOR = 0.09, 95% CI (0.05–0.16)). The odds of getting underweight among children whose mothers had high decision-making power decreased by 93% when compared to children whose mothers had low decision-making power (AOR = 0.07, 95% CI (0.04–0.12)) after controlling for other variables. The proportion of underweight children is higher (63.2%) in mothers who had low decision-making power compared to mothers who had high decision-making power (9.3%) (Table 4).

## 4. Discussion

A quarter (24.6%) of the children were underweight, indicating high magnitude of undernutrition in the study area [11]. This finding is comparable with the national (25%) and regional (25.7%) levels [7] as well as the finding of the study from Lalibela [12]. However, this prevalence was lower compared to the prevalence reported by a number of other studies conducted in developing countries and different parts of Ethiopia [13–20]. The difference might be that this study includes only urban setup, whereas the other studies include also rural areas.

In multivariate logistic analysis, maternal decision-making power, educational status and occupation of mother, monthly income of the household, age and sex of child, and child illness remain significantly associated with underweight.

The odds of getting underweight among children whose mothers had high decision making power decreased by 93% compared to children whose mothers had low decision-making power. This finding was in-line with EDHS 2011 [21], which indicated that children whose mothers participated in decision-making have 0.035 higher units of nutritional status than those whose mothers did not. The finding was also similar to a study from Bangladesh [22], International Food Policy Research Institute's report [23], and Egypt [24], which inferred that women's decision-making power is significant and has positive effect on height-for-age, weight-for-height, and weight-for-age. Mothers with high decision-making power have control within their households of accessing resources, behaving independently, and being able to act in a manner that best promotes the survival and growth of their children [25].

Male children were 1.8 times more likely to be underweight than females. This result is consistent with results of studies conducted in different parts of Ethiopia, Bule Hora [26] and Dollo Ado [27] districts as well as Vietnam [28]. This might be explained by the fact that boys are more influenced by environmental stress than girls [28]; but more prospective cohort studies are warranted.

Increased maternal educational status had a protective effect. The odds of getting underweight among mothers who had diplomas and higher educational statuses decreased by 81% more than mothers who had only secondary education. The finding was consistent with a study conducted in Ethiopia [29, 30]. The possible explanation could be that mothers who had better education had better income and better childcare practice.

Children from families whose monthly income was 4000.00 ETB and below were more likely to be underweight than those who had families with income greater than 4000.00 ETB. This result is in-line with the WHO indicators report, which shows that families with higher monthly per capita income had significantly lower prevalence of underweight children [31], which is also supported by different studies in different parts of Ethiopia like the southern region [27] and Dollo Ado [15]. Wealthy families can get enough food for their children, as a result underweight is unlikely.

Children who did not experience any illness in the last two weeks prior to the survey had a 91% decreased chance of being underweight than those who were ill. Similar studies had also found significant association between the presence of diarrhea within the last two weeks before the survey and underweight in children [15, 26, 32]. As diarrhea depletes the body fluids, children with diarrhea are more likely to lose weight and show low weight for their ages [33].

## 5. Conclusion

The prevalence of undernutrition among the children was found to be high. Maternal decision-making power has significant association with children's weight. Thus, improving mothers' household decision-making autonomy had a significant influence on children's nutritional status.

## Figures and Tables

**Table 1:** Sociodemographic characteristics of respondents in Wolaita Sodo town, southern Ethiopia, 2016.

Variables (*n* = 642)	Frequency (*N*)	Percentage (%)
Age of the children		
6–11 months	63	9.8
12–23 months	175	27.3
24–35 months	165	25.7
36–47 months	132	20.6
48–59 months	107	16.7
Sex of the children		
Male	333	51.9
Female	309	48.1
Educational status		
No formal education	107	16.7
Primary education	287	44.7
Secondary education	98	15.3
Diplomas and higher education	150	23.4
Occupation		
Housewife	306	47.7
GOE/NGOE	208	32.4
Merchant	106	16.5
Others	22	3.4
Household family size		
<5	304	47.4
5+	338	52.6
Average monthly income		
≤2000	275	42.8
2001–4000	264	41.1
>4000	103	16.0

**Table 2:** Prevalence of underweight among children aged 6–59 months in Wolaita Sodo town, southern Ethiopia, 2016.

Variables (*n* = 642)	Frequency (*N*)	Percentage (%)
Normal (WAZ ≥ −2 SD)	412	64.2
Moderate underweight (WAZ ≥ −3 SD & <−2 SD)	126	19.6
Severe underweight (WAZ < −3 SD)	32	5.0
Total of underweight children	158	24.6
Underweight by sex		
Male		
Moderate	70	21.0
Severe	22	6.6
Total	92	27.6
Female		
Moderate	56	18.2
Severe	10	3.2
Total	66	21.4
Underweight by age		
6–11 months	21	33.3
12–23 months	44	25.1
24–36 months	45	27.3
37–47 months	33	25.0
48–59 months	15	14.0

**Table 3:** Household decision-making autonomy of mothers in Wolaita Sodo town, southern Ethiopia, 2016.

Variables (*n* = 642)	Frequency	Percentage
Decision-making on small household purchases		
Respondent alone	272	42.4
Jointly	277	43.1
Husband and another person/someone else	7	1.1
Husband alone	86	13.4
Decision-making on large household purchases		
Respondent alone	44	6.9
Jointly	325	50.6
Husband and another person/someone else	8	1.3
Husband alone	265	41.5
Decision-making on mother and child healthcare		
Respondent alone	141	22.0
Jointly	361	56.2
Husband and another person/someone else	7	1.1
Husband alone	133	20.7
Decision-making on right quantity and type of foods to be given to children		
Respondent alone	401	62.5
Jointly	172	26.8
Husband and another person/someone else	5	0.8
Husband alone	64	10.0
Decision-making on family planning		
Respondent alone	68	10.6
Jointly	505	78.7
Husband and another person/someone else	3	0.5
Husband alone	66	10.3
Decision-making on participating on work to earn money		
Respondent alone	46	7.2
Jointly	440	68.5
Husband and another person/someone else	4	0.6
Husband alone	152	23.7
Decision-making on sick child treatment		
Respondent alone	71	11.1
Jointly	420	65.4
Husband and another person/someone else	4	0.6
Husband alone	147	22.9
Summary of maternal decision-making power matrix		
Low	182	28.3
High	460	71.7

**Table 4:** Bivariate and multivariate analyses of Sociodemographic characteristics of study participants in Sodo town, southern Ethiopia, 2016.

Variables (*n* = 642)	Underweight		COR (95% CI)	AOR (95% CI)
	No (%)	Yes (%)		
Age of the children				
6–11 months	42 (66.7%)	21 (33.3%)	3.07 (1.44–6.53)^*∗*^	2.05 (0.74–5.69)
12–23 months	131 (74.9%)	44 (25.1%)	2.06 (1.08–3.92)^*∗*^	2.30 (0.99–5.32)
24–35 months	120 (72.7%)	45 (27.3%)	2.30 (1.21–4.38)^*∗*^	2.36 (1.03–5.43)^*∗*^
36–47 months	99 (75.0%)	33 (25.0%)	2.04 (1.04–4.01)^*∗*^	2.35 (0.97–5.69)
48–59 months	92 (86.0%)	15 (14.0%)	1	1
Sex				
Male	241 (72.4%)	92 (27.6%)	1.4 (.98–2.02)	1.82 (1.07–3.09)^*∗*^
Female	243 (78.6%)	66 (21.4%)	1	1
Educational status				
No formal	52 (48.6%)	55 (51.4%)	9.5 (4.95–18.31)^*∗*^	0.88 (0.23–3.33)
Primary	208 (72.5%)	79 (27.5%)	3.4 (1.89–6.19)^*∗*^	0.42 (0.13–1.43)
Secondary	89 (90.8%)	9 (9.2%)	0.91 (0.38–2.17)	0.19 (0.05–0.73)^*∗*^
Diplomas & higher education	135 (90.0%)	15 (10.0%)	1	1
Occupation				
GOE/NGOE	192 (92.3%)	16 (7.7%)	1	1
Housewife	197 (64.4%)	109 (35.6%)	6.6 (3.79–11.64)^*∗*^	3.26 (1.06–9.99)^*∗*^
Merchant	80 (75.5%)	26 (24.5%)	3.9 (1.99–7.66)^*∗*^	5.29 (1.58–17.70)^*∗*^
Daily laborer	10 (62.5%)	6 (37.5%)	7.2 (2.32–22.36)^*∗*^	2.04 (0.31–13.43)
Others	5 (83.3%)	1 (16.7%)	2.4 (0.26–21.81)	2.52 (0.15–43.18)
Average monthly income				
≤2000	182 (66.2%)	93 (33.8%)	12.6 (4.51–35.44)^*∗*^	2.38 (0.85–6.69)
2001–4000	203 (76.9%)	61 (23.1%)	7.4 (2.63–21.04)^*∗*^	4.16 (1.57–11.05)^*∗*^
>4000	99 (96.1%)	4 (3.9%)	1	1
Time of introducing complementary food				
Before 6 months	79 (61.2%)	50 (38.8%)	2.4 (1.57–3.59)^*∗*^	1.06 (0.57–1.98)
6–8 months	405 (78.9%)	108 (21.2%)	1	1
Child illness in the last two weeks				
No	445 (84.8%)	80 (15.2%)	0.09 (0.06–0.14)^*∗*^	0.09 (0.05–0.16)^*∗∗*^
Yes	39 (33.3%)	78 (66.7%)	1	1
DDS				
Poor DDS (1–3)	71 (53.8%)	61 (46.2%)	4.50 (2.49–7.96)^*∗*^	1.54 (0.65–3.62)
Med DDS (4–6)	304 (80.0%)	76 (20.0%)	1.30 (0.76–2.21)	0.70 (0.34–1.45)
Good DDS (7+)	109 (83.8%)	21 (16.2%)	1	1
Maternal decision-making power				
Low	67 (36.8%)	115 (63.2%)	1	1
High	417 (90.7%)	43 (9.3%)	0.06 (0.04–0.09)^*∗*^	0.07 (0.04–0.12)^*∗∗*^

^*∗*^Significant at *P* value < 0.05; ^*∗∗*^significant at *P* value < 0.001.

## Abbreviations

DDs: Dietary diversity score
EDHS: Ethiopian Demographic and Health Survey
HHs: Households
SNNPR: Southern Nation National and People Region
WAZ: Weight for age* Z* score
WHO: World Health Organization.

## Data Access

The datasets analyzed during the current study are available with the corresponding author on reasonable request.
